# A critical evaluation of parametric models for predicting faecal indicator bacteria concentrations in greywater

**DOI:** 10.1016/j.mran.2024.100297

**Published:** 2024-04

**Authors:** Émile Sylvestre, Michael A. Jahne, Eva Reynaert, Eberhard Morgenroth, Timothy R. Julian

**Affiliations:** a Eawag, Swiss Federal Institute of Aquatic Science and Technology, 8600 Dübendorf, Switzerland; b Sanitary Engineering, Delft University of Technology, Stevinweg 1, 2628 CN, Delft, the Netherlands; c U.S. Environmental Protection Agency, 26 W. Martin Luther King Dr., Cincinnati OH 45268, United States; d ETH Zürich, Institute of Environmental Engineering, 8093 Zürich, Switzerland; e Swiss Tropical and Public Health Institute, CH-4123 Allschwil, Switzerland; f University of Basel, 4055 Basel, Switzerland

**Keywords:** Greywater reuse, *Escherichia coli*, Poisson lognormal distribution, Water Treatment, Health Risks, Quantitative Microbial Risk Assessment (QMRA)

## Abstract

Greywater reuse is a strategy to address water scarcity, necessitating the selection of treatment processes that balance cost-efficiency and human health risks. A key aspect in evaluating these risks is understanding pathogen contamination levels in greywater, a complex task due to intermittent pathogen occurrences. To address this, faecal indicator organisms like *E. coli* are often monitored as proxies to evaluate faecal contamination levels and infer pathogen concentrations. However, the wide variability in faecal indicator concentrations poses challenges in their modelling for quantitative microbial risk assessment (QMRA). Our study critically assesses the adequacy of parametric models in predicting the variability in *E. coli* concentrations in greywater. We found that models that build on summary statistics, like medians and standard deviations, can substantially underestimate the variability in *E. coli* concentrations. More appropriate models may provide more accurate estimations of, and uncertainty around, peak *E. coli* concentrations. To demonstrate this, a Poisson lognormal distribution model is fit to a data set of *E. coli* concentrations measured in shower and laundry greywater sources. This model estimated arithmetic mean *E. coli* concentrations in laundry waters at approximately 1.0E + 06 MPN 100 mL^−1^. These results are around 2.0 log_10_ units higher than estimations from a previously used hierarchical lognormal model based on aggregated summary data from multiple studies. Such differences are considerable when assessing human health risks and setting pathogen reduction targets for greywater reuse. This research highlights the importance of making raw monitoring data available for more accurate statistical evaluations than those based on summary statistics. It also emphasizes the crucial role of model comparison, selection, and validation to inform policy-relevant outcomes.

## 1. Introduction

Greywater reuse offers an opportunity for water conservation at the scale of a household or a building. However, monitoring pathogen concentrations in greywater, particularly when generated by small populations, presents challenges. These challenges primarily originate from the intermittent occurrence of pathogens and their potentially low concentrations in greywater. The lack of reliable pathogen concentration data poses an obstacle in conducting health risk assessments for greywater reuse. As an alternative to the direct measurements of pathogens, monitoring of faecal indicators, such as *E. coli*, somatic coliphages, and *Clostridium perfringens* can inform on the level of faecal contamination in greywater. Mathematical models integrating faecal indicator and epidemiological data have been developed to predict enteric pathogen concentrations in water sources characterized by low pathogen occurrences and concentrations. These models predict the concentration of pathogens in water by considering (i) community infection rates to infer the proportion of the population shedding the pathogen, (ii) the duration of pathogen shedding, (iii) the concentration of a selected faecal indicator in the water; and (iv) the densities of the faecal indicator and the pathogen in faeces. This method has been applied to various water sources, including recreational water (Gerba, 2000; Soller et al., 2010), stormwater (Petterson et al., 2016), and greywater (Barker et al., 2013; Jahne et al., 2017; Ottoson and Stenström, 2003).

The accuracy of these models relies on proper estimates of the model parameters. The estimation of faecal indicator concentrations in water is of particular significance, as these are directly proportional to the pathogen concentration during a contamination event. In specific greywater sources, such as greywater originating from laundry and showers, faecal indicator concentrations can vary widely, often characterized by a high proportion of non-detects and occasionally very high concentrations in some samples. For instance, *E. coli* concentrations can range from undetectable to over 1.0E+06 most probable number (MPN) per liter (O’Toole et al., 2012). Achieving accurate modelling of faecal indicator concentrations in such variable greywater sources is a challenging yet important task. Complicating this issue is a common lack of site-specific faecal indicator monitoring data, necessitating the use of literature values in risk assessment. This is further complicated by the literature’s use of summary statistics to report on *E. coli* concentrations instead of providing raw data. The use of summary statistics is insufficient for in-depth statistical analysis. Moreover, the uncertainty in faecal indicator concentrations associated with summary statistics (confidence interval) is typically not conveyed, potentially yielding faecal indicator concentration estimates that may seem overly precise. While approximation techniques to incorporate these summary statistics into parametric models have been proposed (Jahne et al., 2017), the considerable variability in faecal indicator concentrations and the omission of the uncertainty surrounding the summary statistics raise concerns about the reliability of these methods.

Within this modelling framework, inaccuracies in characterizing the distributions of faecal indicator concentrations can affect predictions of the distributions of pathogen concentrations. These predictions are critical to ensuring that greywater treatment sufficiently protects human health. The level of treatment should be guided by risk-based evidence on the minimum log reduction targets (LRTs), informed by quantitative microbial risk assessment (QMRA) (Jahne et al., 2023; Schoen et al., 2017; Shi et al., 2018). These LRTs, in turn, guide the selection of water treatment technologies to achieve the required pathogen reduction (Pecson et al., 2022). Consequently, the reliability of greywater treatment strategies, and thus the protection of public health, relies on accurate estimates of faecal indicator concentrations.

The objectives of this study are two-fold. Firstly, to critically analyze approximation methods to evaluate the variability in faecal indicator concentrations based on summary statistics from primary studies. This includes examining the potential pitfalls of these methods, especially when dealing with high variability in concentrations from original data sets, and the challenges in combining summary statistics for meta-analysis. Secondly, to propose candidate parametric models to describe the variability and uncertainty in concentrations of faecal indicators in freshly-collected greywater when raw data are available in primary studies.

## 2. Materials and methods

Of the greywater quality studies reviewed by Jahne et al. (2017), only O’Toole et al. (2012) provided sufficient raw data for parametric modelling. O’Toole et al. (2012) assessed *E. coli* concentrations in laundry wash, rinse, and shower/bath waters across 93 households. This spatial variability in *E. coli* concentrations was analyzed using two distinct modelling approaches: the approximation method used by Jahne et al. (2017) and parametric modelling of raw data. This comparative analysis aims to assess the capacity of these two approaches to reflect the variability in *E. coli* concentrations in greywater sources when raw data are available.

### 2.1. Estimation of lognormal parameters using summary statistics

The method proposed by Jahne et al. (2017) to describe the concentration of *E. coli* in greywater sources involves deriving the parameter values of μ and σ of a lognormal (LN) distribution using summary statistics. The value of σ is approximated from concentrations of *E. coli* reported in the study by O’Toole et al. (2012) using the formula $\sigma \approx \sqrt{\ln\left(\text{SD/Median}\right)}$, where SD and Median are the sample standard deviation and median. The between-study heterogeneity (i.e., variation in the value of μ across studies) is accounted for using a PERT distribution, where the mode for μ is estimated based on the sample median from O’Toole et al. (2012), and the minimum and maximum values of μ are approximated from sample arithmetic means reported in Rose et al. (1991), Friedler (2004) and Jefferson et al. (2004) using the formula $\mu \approx \ln\left(\text{Mean}\right)-\sigma^{2}\text{/}2$, where σ is derived from O’Toole et al. (2012) data (Table 1). We will subsequently refer to this model as a “hierarchical LN-PERT model.”

### 2.2. Derivation of the standard deviation approximation for a lognormal distribution

The calculations of σ through the approximation $\sigma \approx \sqrt{\ln\left(\text{SD/Median}\right)}$ is rooted in statistical principles governing the lognormal distribution. This section elucidates how this approximation is derived from the mathematical properties of this distribution.

For a lognormally-distributed *E. coli* concentration c, the median is given by $e^{\mu }$, where μ is the mean of $\ln\left(c\right)$. The SD of c can be calculated via:

(3) $$\text{SD}=\sqrt{\left(e^{\sigma^{2}}-1\right)e^{2\mu +\sigma^{2}}}$$

Since the median of c is $e^{\mu }$, the expression $\frac{\text{SD}}{\text{Median}}$ simplifies to:

$$\frac{\text{SD}}{e^{\mu }}=\sqrt{\left(e^{\sigma^{2}}-1\right)e^{\sigma^{2}}}$$

Logarithmically transforming this yields:

$$\ln\left(\frac{\text{SD}}{e^{\mu }}\right)=\frac{1}{2}\ln\left(\left(e^{\sigma^{2}}-1\right)e^{\sigma^{2}}\right)$$

Square rooting both sides, we obtain:

$$\sqrt{\ln\left(\frac{SD}{e^{\mu }}\right)}=\sqrt{\frac{1}{2}\ln\left(\left(e^{\sigma^{2}}-1\right)e^{\sigma^{2}}\right)}$$

If we assume $e^{\sigma^{2}}-1$ is close enough to $e^{\sigma^{2}}$ (an approximation that holds for small σ), then:

$$\sqrt{\ln\left(\frac{SD}{e^{\mu }}\right)}\approx \sigma$$

Or reformatted to match the original equation:

(4) $$\sigma \approx \sqrt{\text{In}\left(\frac{\text{SD}}{\text{Median}}\right)}$$

To assess the reliability of this σ approximation method to capture the variability in concentrations, we ran 100 simulations for each true σ value, ranging from 0.1 to 6.0 at 0.1 intervals. This extensive range of σ values was selected to encompass lognormal distributions ranging from thin tails to very heavy tails. Each simulation generated 1,000 lognormally distributed random variables with μ set to 0. The sample median and SD were computed for these variables, and Eq. (4) was applied to estimate σ. Our approach assumes that n=1000 fully captures the variability in concentrations.

### 2.3. Bayesian inference of mixed Poisson distributions

Of the studies utilized by Jahne et al. (2017), only O’Toole et al. (2012) provided raw data for parametric distribution fitting. These data sets include non-detects and exhibit high variability, with concentrations spanning several orders of magnitude. In such cases, discrete parametric models, such as mixed Poisson distributions (Haas et al., 2014), are recommended for statistical inference (Chik et al., 2018). O’Toole et al. (2012) data sets were analyzed using three candidate models commonly used in quantitative microbiology (El-Shaarawi et al., 1981; Masago et al., 2006; Sylvestre et al., 2020): Poisson Gamma distribution (PGA), Poisson lognormal distribution (PLN), and Poisson Lomax distribution (PLO).

Mixed Poisson models combine the Poisson distribution, which derives sample concentration from the microorganism count and the volume analyzed, with a continuous distribution that captures the variability in concentrations across samples (Haas et al., 2014; WHO, 2016).

The Poisson distribution is defined as:

(5) $$P(k;c,V)=\frac{cV\cdot e^{-cV}}{k!}$$

Here, k is the *E. coli* count, and c×V is the expected value, with c being the *E. coli* concentration and V the volume of the water sample. Since individual dilutions and the number of positive/negative wells per sample were not provided by O’Toole et al. (2012), we assumed a volume of 100 mL for all samples and used this assumed volume to infer counts from concentrations.

The probability density function (PDF) of the Gamma distribution is:

(6) $$P(c;\alpha ,\beta )=\frac{\beta^{\alpha }}{\text{Γ}\left(\alpha \right)}c^{\alpha -1}e^{-\beta c}$$

where α and β are the shape and rate parameters, respectively. The PDF of the lognormal distribution is:

(7) $$P(c;\mu ,\sigma )=\frac{1}{c\;\sigma \sqrt{2\pi }}\exp\left(-{\frac{\left(\text{In}c-\mu \right)}{2\sigma^{2}}}^{2}\right)$$

In this expression, the natural logarithm of c is normally distributed with mean μ and variance $\sigma^{2}$. The PDF of the Lomax distribution is formulated as:

(8) $$P(c;\alpha ,\lambda )=\frac{\alpha }{\lambda }{\left[1+\frac{c}{\lambda }\right]}^{-\left(\alpha +1\right)}$$

where α and λ are shape and scale parameters, respectively.

Inference was performed using a Bayesian framework, adopting non-informative priors as detailed in Sylvestre et al. (2021). The analysis was conducted via rjags (v4.14) (Plummer, 2012) within R (v4.3.1). Four Markov chains were run for 1 × 10^5^ iterations, following an initial burn-in of 10^4^ iterations. Chain convergence was monitored using the Brooks–Gelman–Rubin scale reduction factor (Gelman and Shirley, 2011). To ensure a comprehensive exploration of posterior distributions, the effective sample size (ESS), which adjusts the sample size for auto-correlation within the chains, was evaluated (Kass et al., 1998). The R code is provided on GitHub, and the URL to access it is https://tinyurl.com/msvf27pm.

### 2.4. Model comparison

A visual comparison of the *E. coli* concentration predictions was carried out by overlaying complementary cumulative distribution functions (CCDFs) of the lognormal distribution derived from summary statistics, the Gamma distribution of the PGA, the lognormal distribution of the PLN, and the Lomax distribution of the PLO against the reported *E. coli* concentrations. This overlay provided a graphical representation of the distributional fit to the observed data. For data sets where the PGA and the PLN had a similar fit, the deviance information criterion (DIC) (Spiegelhalter et al., 2002) was computed to compare the goodness of fit of Gamma and lognormal distributions. To apply this procedure, non-detects were adjusted to a detection limit of 1 MPN 100 mL^−1^. Lower DIC values indicate a superior model. A practical guideline for DIC comparison suggests that models within a 1–2 range of the “best” model merit attention, whereas those with a 3–7 difference show significantly less support (Spiegelhalter et al., 2002).

Additionally, arithmetic mean *E. coli* concentrations predicted by each model were compared. The arithmetic mean was selected for this analysis as it is the appropriate summary statistic for characterizing microbial risk in QMRA (Haas, 1996). The comparison included arithmetic mean *E. coli* concentrations as estimated by (i) the lognormal distribution derived from summary statistics of O’Toole et al. (2012), (ii) the PGA, PLN, and PLO distributions adjusted to reported data from O’Toole et al. (2012), (iii) the hierarchical lognormal model from Jahne et al. (2017), which synthesize summary statistics from multiple studies.

## 3. Results and discussion

### 3.1. Limitations of the approximation method to predict variations in E. coli concentrations from summary statistics

**Fig. 1.**
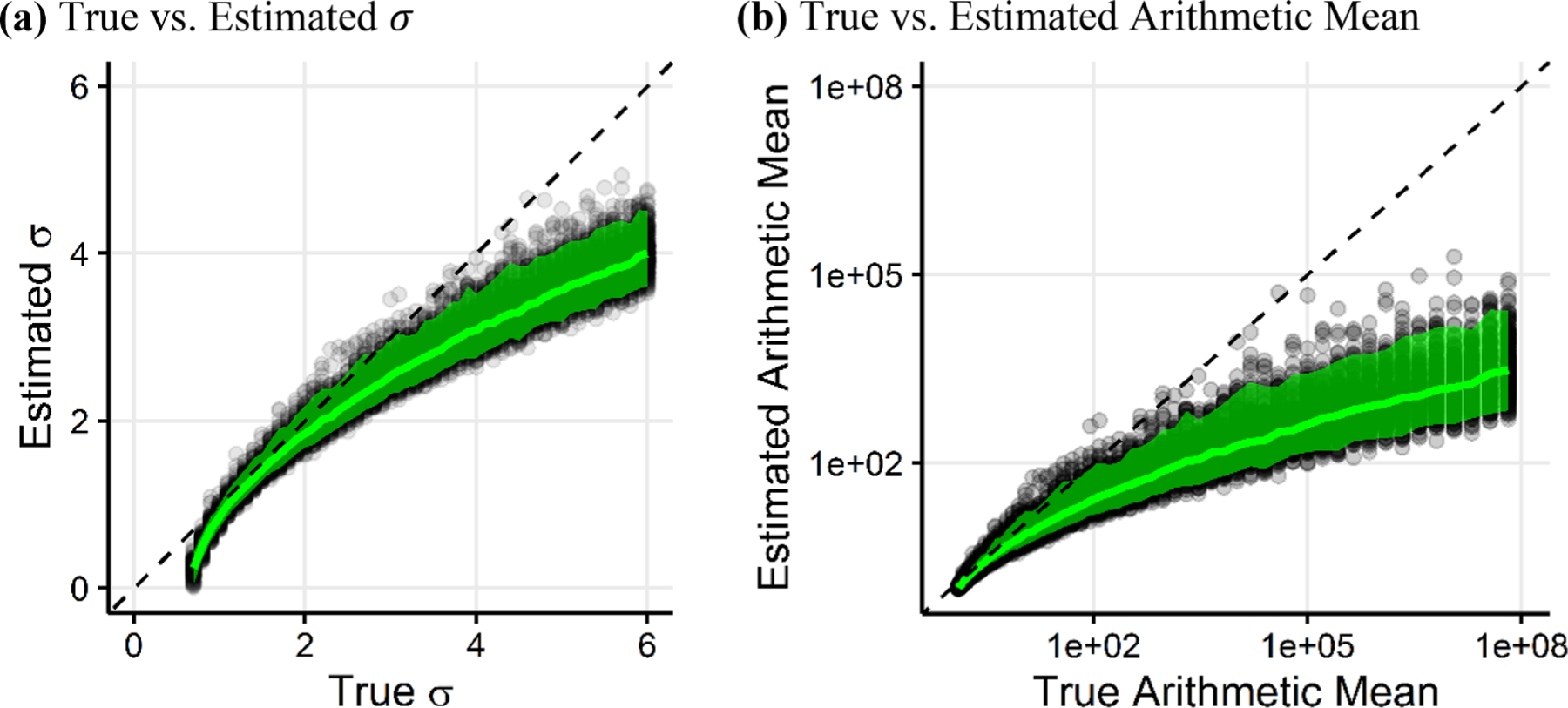
Comparison of lognormal distribution parameters. (a) True standard deviation $\left(\sigma \right)$ versus estimated σ using the approximation method outlined in Eq. (4). (b) True arithmetic mean versus estimated arithmetic mean, with σ approximated using Eq. (4). The green lines indicate the median value of the estimated σ and estimated arithmetic mean. The shaded areas represent the intervals bounded by the 2.5 and 97.5 quantiles of the estimated σ and estimated arithmetic mean.

The relationship between true and estimated σ value approximated from Eq. (4) using the sample median and standard deviation for a sample size of n=1000 shows that for low $\sigma \left(<2.0\right)$, the method is reasonably accurate (Fig. 1a). However, when the true σ exceeds 2.0, the approximation method underestimates the true σ. Underestimating the true σ results in a severe underestimation of the arithmetic mean of the lognormal distribution, particularly for high σ values (Fig 1b). Fig. 1a also demonstrates that the approximation approach fails at low true σ values; this occurs because the approximation cannot be computed when the sample median exceeds the sample standard deviation.

The value of σ estimated with the Poisson lognormal distribution fitted to O’Toole et al. (2012) data are 5.39 for laundry wash, 4.96 for laundry rinse, and 3.22 for shower/bath (Table 2), which is out of the domain of application of the approximation method. The value of σ estimated with the sample median and sample standard deviation of O’Toole et al. (2012) data are 3.61 for laundry wash, 3.20 for laundry rinse, and 1.88 for shower/bath (Table 1).

**Fig. 2.**
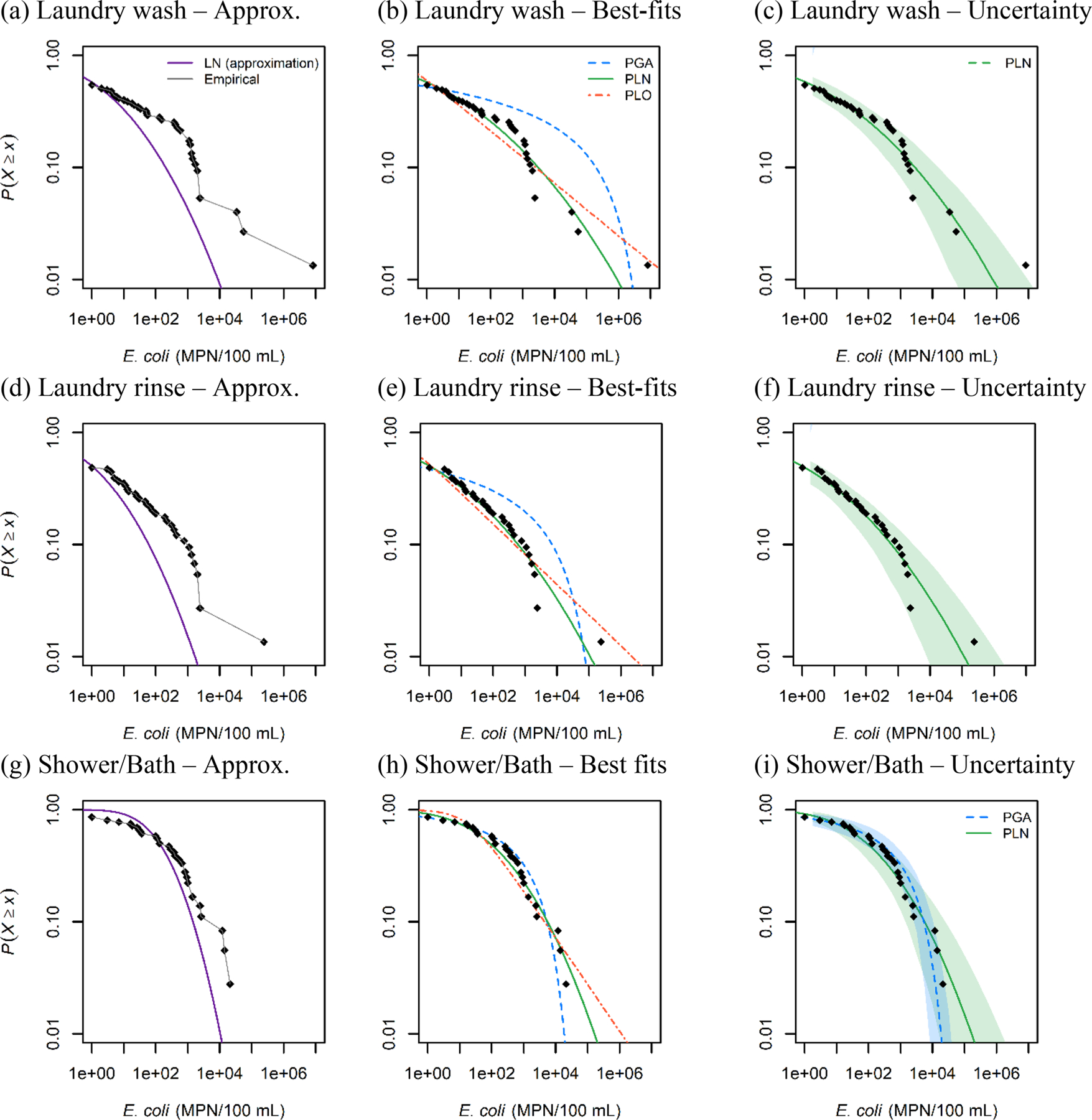
Complementary cumulative distribution function (CCDF) of E. coli concentrations (MPN 100 mL^−1^) in household water sources reported by O’Toole et al. (2012). Left panels (a, d, g) compare empirical distributions to lognormal distributions fitted using the approximation method based on the sample median and standard deviation. Center panels (b, e, h) show best-fits for Poisson gamma (PGA), Poisson lognormal (PLN), and Poisson Lomax (PLO) distributions. Right panels (c, f, i) show the PLN (and the PGA for shower/bath water) with a 95 % uncertainty interval.

The approximation of σ using summary statistics underestimates high *E. coli* concentrations from the data sets, as shown by overlaying CCDFs of the lognormal distribution on the observations (Fig. 2). The maxima are underestimated by approximately 3.0 log_10_ units for laundry wash water, 2.0 log_10_ units for laundry rinse water, and 1.0 log_10_ units for shower/bath water (Fig. 2), potentially resulting in a significant underestimation of the health risks associated with these water sources.

### 3.2. Mixed Poisson distributions to predict variations in E. coli concentrations from raw data

When comparing candidate mixed Poisson models, the PGA distribution underestimates high *E. coli* concentrations for laundry wash and rinse samples (Fig. 2). While the PLO distribution predicts high concentrations well, implementing this model is impractical because its arithmetic mean is undefined for the three data sets, as α≤1 (Table 2). It is undefined due to the nature of its probability density function. When α≤1, the tail of the distribution is so heavy that the arithmetic mean is not a finite number. Therefore, the PLN distribution emerged as the favored model for this data set.

For shower/bath water, both PGA and PLN distributions showed comparable fits. However, the upper tail of the PLN predicts higher concentrations than the PGA. The DIC of the lognormal fit of 513 was significantly lower than the DIC of the Gamma fit of 520, suggesting that the PLN provides a better fit than the PGA for these data. It is important to note that this comparison involved adjusting the two non-detects of this data set to a detection limit of 1 MPN 100 mL^−1^. In scenarios with a more significant proportion of non-detects, a more advanced information criterion should be employed to compare mixed Poisson distributions directly. The marginal DIC (Quintero and Lesaffre, 2018) effectively compared mixed Poisson distributions for *Cryptosporidium* count data monitored in drinking water sources (Sylvestre et al., 2021). However, applying the mDIC in our study did not yield successful outcomes, likely because of the magnitude of the variability. Investigating information criteria suitable for these types of datasets could enhance the comparison of models.

**Fig. 3.**
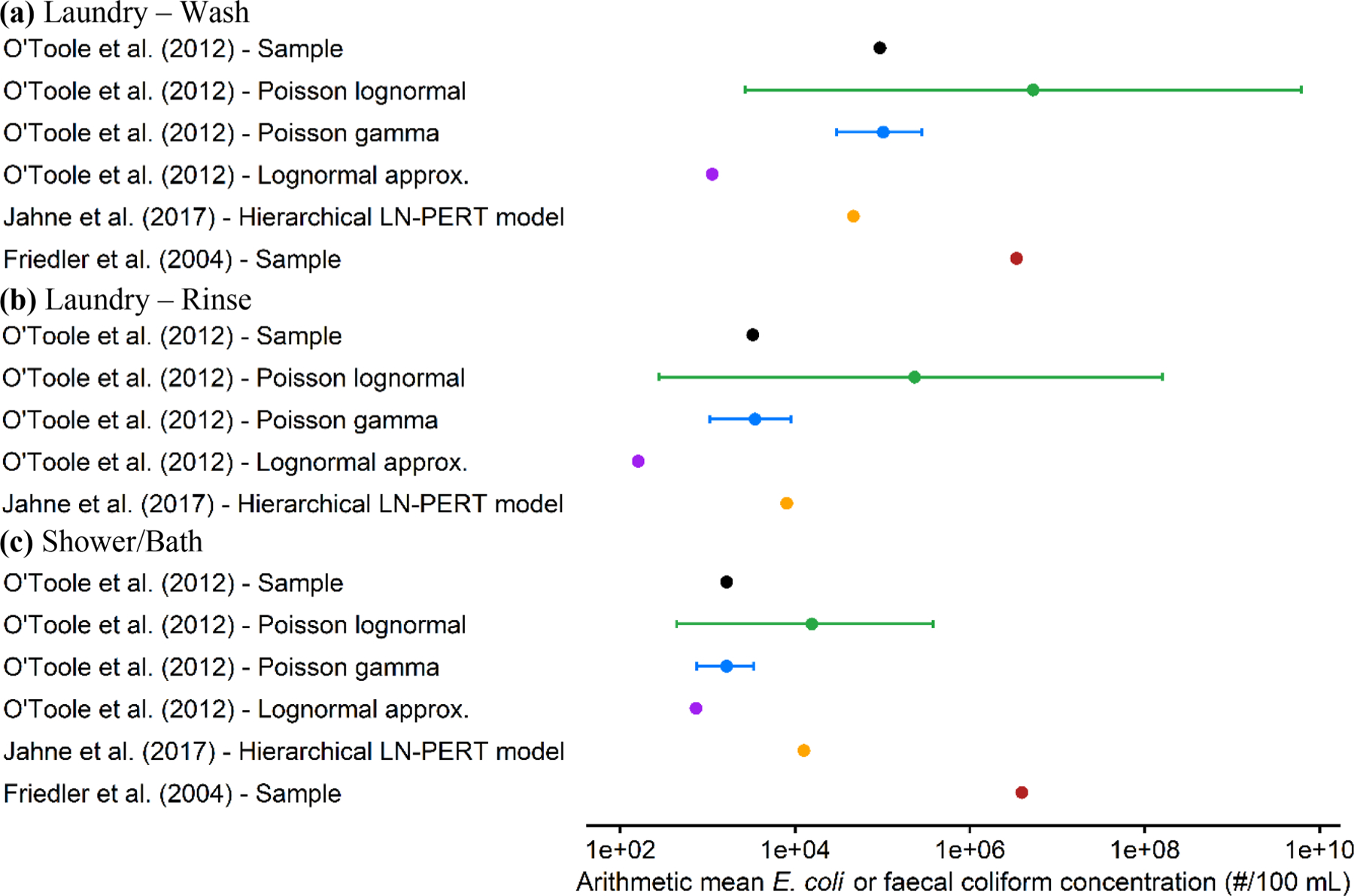
Arithmetic mean *E. coli* or fecal coliform concentration in household greywater sources reported by O’Toole et al. (2012) and Friedler (2004). Data points are stratified into three categories—laundry wash (a), laundry rinse (b), and shower/bath (c)—with corresponding estimates derived from the sample mean, Poisson lognormal, Poisson gamma, lognormal distributions fitted using the approximation method based on the sample median and standard deviation, the hierarchical lognormal model from Jahne et al. (2017), and the sample mean fecal coliform concentration from Friedler (2004). The 95 % credible intervals are illustrated for the Poisson lognormal and Poisson gamma. This comparison excludes mean values from Rose et al. (1991) and Jefferson et al. (2004) due to possible reporting of geometric means instead of arithmetic means.

Empirical arithmetic mean *E. coli* concentrations for laundry wash, rinse, and shower/bath waters, calculated by replacing non-detects by concentrations of zero MPN 100 mL^−1^, are 1.1E+05, 3.4E+03, and 1.7E+03 MPN 100 mL^−1^, respectively (Fig. 3). These values are notably lower than those predicted by the PLN distribution, indicating a significant influence of the upper tail of this distribution on the arithmetic mean. Truncating the PLN distribution to eliminate high concentrations that occur with very low exceedance probabilities (e.g., < 0.001) could make the predicted arithmetic means more closely match the empirical arithmetic means. Nevertheless, Friedler (2004) documented high sample arithmetic mean faecal coliform concentrations of 4.0E+06 colony forming unit (CFU) 100 mL^−1^ in laundry and shower/bath waters (Fig. 3). For laundry water, this reported arithmetic mean faecal coliform concentrations are in the same range as the arithmetic mean *E. coli* concentration predicted by the PLN, suggesting that the upper tail of the PLN distribution can predict peak concentrations in these sources. It is important to recognize that *E. coli* represents a fraction of faecal coliforms; nonetheless, the extent of this fraction is unlikely to substantially alter the outcome. As one example, Garcia-Armisen et al. (2007) found a *E. coli*:fecal coliform ratio of 0.77 in various contaminated freshwater samples, signifying that, on average, 77 % of fecal coliforms are *E. coli*. Therefore, these results indicate that the empirical mean may not be a reliable summary estimate for heavy-tailed distributions because it can substantially underestimate the “true” mean, especially at small sample size.

### 3.3. Comparison of the Poisson lognormal model predictions with the hierarchical lognormal-PERT model

For laundry wash and laundry rinse water, arithmetic mean *E. coli* concentrations predicted by the best-fit parameters of the PLN distribution are 2.1 and 1.5 log_10_ units higher, respectively, than those predicted by the hierarchical LN-PERT model from Jahne et al. (2017) (Fig. 3). For shower/bath water, the PLN distribution predicts an arithmetic mean concentration consistent with the hierarchical LN-PERT model. As described in Section 2.1, the hierarchical LN-PERT model uses approximation formulas to calculate lognormal parameters from sample statistics. σ was approximated from *E. coli* concentrations reported by O’Toole et al. (2012), and a PERT distribution was used to address the between-study heterogeneity (i.e., variations in μ across various studies).

As discussed in Section 3.1, Jahne et al. (2017) approach to approximate σ from sample statistics underestimates the variability, partly explaining the differences between arithmetic means of the hierarchical LN-PERT model and the PLN of 2.1-log_10_ for laundry wash water, of 1.4-log_10_ for laundry rinse water. Additionally, the mean values reported in studies by Rose et al. (1991) and Jefferson et al. (2004) appear to be calculated on a logarithmic scale, implying they are geometric means rather than the arithmetic means required for μ approximation. This misunderstanding likely further contributes to the observed differences.

A key difference between the hierarchical LN-PERT model and the PLN is that the former does not provide information on the confidence associated with the arithmetic mean. This arises because the current hierarchical LN-PERT model does not separate the parametric uncertainty of μ from the variability, as it is computed through a single-dimension Monte Carlo simulation. To separate uncertainty from variability, more sophisticated methods, like second-order Monte Carlo simulation, have been advocated for QMRA (Haas et al., 2014; Pouillot and Guillier, 2020).

For a comprehensive combination of data sets from multiple studies, the development of a statistical meta-analysis approach is necessary. The current hierarchical LN-PERT model assumes that the σ estimate derived from O’Toole et al. (2012) data applies to other data sets, which might not be true. High faecal indicator concentrations, particularly in data sets with heavy tails, can increase the sample variance and, consequently, the estimated σ. The magnitude and frequency of such high concentrations will likely vary among different sites or studies, influenced by distinct human behaviors and demographic variables. For example, Rose et al. (1991) documented that households with young children generated greywater with faecal coliform concentrations reaching 1.0E+06 CFU 100 mL^−1^, contrasting sharply with adult-only households generating concentrations around 1.0E+02 CFU 100 mL^−1^.

Additionally, it is essential to quantify the standard error of lognormal parameter values from each study for an accurate statistical meta-analysis. Ignoring standard errors may result in inappropriate weighting, giving equal importance to less precise studies as to more precise ones.

### 3.4. Selection of a model for simulating pathogen concentrations in greywater

Our study relies only on the data set from O’Toole et al. (2012), opting against synthesizing summary statistics from multiple studies. This decision is due to inappropriate data reporting that prevents appropriate statistical analyses for risk assessment. The implication of this re-analysis of *E. coli* concentrations on the definition of LRTs and the selection of treatment technologies is explored in Reynaert et al. (2024).

Additional data collection, particularly from laundry wash and rinse waters, is crucial to assess the generalizability of the PLN distribution fitted to O’Toole et al. (2012) data. Relying solely on this single study to characterize local conditions may result in inaccuracies in risk predictions. O’Toole et al. (2012) provide a snapshot of *E. coli* concentration variations across multiple households, each represented by a single sample collected by the residents. This approach does not capture potential temporal variations within the same household. Considering that our fitted distributions are heavily influenced by a few peaks, identifying and mitigating factors leading to high contamination events in single households could lower the arithmetic mean and reduce its associated uncertainty, thereby reducing the estimated health risks and the corresponding need for treatment. Furthermore, sampling of household water sources is typically carried out by residents and has to be done for various types of installation. The development of procedures to standardize sampling methods could, therefore, be beneficial to ensure consistency.

### 3.5. Other sources of uncertainty for the prediction of pathogen concentrations in greywater

In this study, the level of faecal contamination in greywater sources was evaluated using reported *E. coli* concentrations in freshly-collected greywater. The likelihood of *E. coli* growth impacting results is thus minimal. This claim is supported by Khalaphallah and Andres (2012), who demonstrate that the *E. coli* concentration in bathroom greywater stored at a temperature of 23 °C increases by approximately 0.5-log_10_ after about 120 h. Therefore, our model’s predictions are based on scenarios where *E. coli* concentrations reflect initial fecal contamination rather than subsequent microbial growth. Although O’Toole et al. (2012) targeted freshly collected greywaters (i.e., before storage or treatment), any environmental or engineered barriers between the faecal sources and the sampling point could exert differential effects on the proxy *E. coli* and the enteric pathogen of interest. For laundry wash and rinse waters, the fate of *E. coli* between the faecal source (i.e., pathogens in/on articles of laundry) and the sampling point (the laundry wash or rinse waters) is not necessarily the same as the fate of all enteric pathogens (Reynolds et al., 2022). A laboratory seeding study comparing the impacts of laundry detergents, additives like chlorine bleach, and water temperature on the fate of faecal indicators and pathogens during laundry processes would help identify the most representative faecal indicator for enteric pathogens of interest.

The current mathematical model used to simulate enteric pathogen concentrations in greywater (Jahne et al., 2017) relies on *E. coli* densities in feces, measured in CFU g^−1^ (Feachem et al., 1983), aligning with greywater concentrations measured in CFU L^−1^ reported by O’Toole et al. (2012). The use of culture-based detection methods may underestimate *E. coli* due to the potential for viable but nonculturable (VBNC) *E. coli*. Greywater’s composition, characterized by the presence of antimicrobials, soaps, surfactants, and elevated pH, may influence the proportion of VBNC *E. coli* relative to faecal samples. If there is an elevated proportion of VBNC in greywater, the fecal load in QMRA models may be underestimated. Employing molecular methods to quantify faecal indicators/markers in both feces and greywater may reduce the uncertainty introduced by the potential for VBNC organisms.

## 4. Conclusions

- The variability in *E. coli* concentrations in greywater sources can be substantial. The assessment of these variations can influence the estimation of public health risks and the required level of treatment before reuse. Therefore, rigorous methods for model comparison, selection, and validation are crucial to ensure accurate predictions of *E. coli* concentrations.
- Of the models tested, the Poisson lognormal distribution was identified as the most suitable model to predict peak *E. coli* concentrations in laundry and showering greywater from a previously published sampling campaign. This model estimated arithmetic mean concentrations of *E. coli* in laundry wash and rinse waters to be about 1.0E+06 MPN 100 mL^−1^, which is about 2.0 log_10_ units higher than those predicted by the hierarchical lognormal-PERT model proposed by Jahne et al. (2017).
- The estimated arithmetic mean of the Poisson lognormal distribution exhibits considerable uncertainty because of its sensitivity to a small number of peak *E. coli* concentrations. Additional data collection, particularly from laundry wash and rinse waters, is crucial to assess the generalizability of the Poisson lognormal distribution and investigate the factors contributing to such high concentrations.
- To develop meta-analysis models to predict faecal indicator concentrations in greywater, primary studies should report raw data rather than solely summary statistics. This approach would enable more accurate modelling and facilitate the comparison and synthesis of data across various sites and studies, for which additional characterization is needed.

## Supplementary Material

Supplementary Materials

## Figures and Tables

**Table 1:** Method and data used by Jahne et al. (2017) to estimate values of the lognormal parameters μ and σ describing the variability in concentrations of *Escherichia coli* in colony forming unit (CFU) or most probable number (MPN) 100 mL^−1^ in greywater from various household sources. Jahne et al. (2017) used a minimum μ, a mode of μ, and a maximum μ as parameters of a PERT distribution to account for the parametric uncertainty in μ.

Lognormal parameter	Laundry wash	Laundry rinse	Shower/Bath
minμ	−1.70 (Rose et al., 1991)	−1.90 (Rose et al., 1991)	2.65 (Jefferson et al., 2004)
modeμ	0.69 (O’Toole et al., 2012)	0.00 (O’Toole et al., 2012)	4.87 (O’Toole et al., 2012)
maxμ	10.08 (Friedler, 2004)	10.08 (Friedler, 2004)	13.43 (Friedler, 2004)
σ	3.61 (O’Toole et al., 2012)	3.20 (O’Toole et al., 2012)	1.88 (O’Toole et al., 2012)

**Table 2:** Best-fit parameter values for the Poisson Gamma (PGA), Poisson lognormal (PLN), and Poisson Lomax (PLO) distributions adjusted to *E. coli* concentrations in greywater from household water sources reported by O’Toole et al. (2012). The 95 % credible interval of the parameter values is provided for the PLN.

Water source	Poisson Gamma		Poisson lognormal		Poisson Lomax	
	α	β	μ	σ	α	λ
Laundry wash	0.05	4.4E-07	1.08 (−0.37, 2.46)	5.39 (4.28, 6.78)	0.23	5.39
Laundry rinse	0.06	1.6E-05	0.04 (−1.40, 1.39)	4.96 (3.83, 6.35)	0.27	0.09
Shower/bath	0.22	1.2E-04	4.49 (3.37, 5.57)	3.22 (2.46, 4.16)	0.41	17.4

## Data Availability

I have shared the link to my code as the Attached File step.
